# Integrated Amplitude and Phase Monitor for Micro-Actuators

**DOI:** 10.3390/mi13081360

**Published:** 2022-08-20

**Authors:** Sandra Nicole Manosalvas-Kjono, Ronald Quan, Olav Solgaard

**Affiliations:** Department of Electrical Engineering, Stanford University, Stanford, CA 94305, USA

**Keywords:** combdrive, phase, open-loop, resonance, sensor, capacitance, MEMS, multiple-frequency bandpass filters, multiple-frequency demodulators

## Abstract

Micro-actuators driven on resonance maximize reach and speed; however, due to their sensitivity to environmental factors (e.g., temperature and air pressure), the amplitude and phase response must be monitored to achieve an accurate actuator position. We introduce an MEMS (microelectromechanical system) amplitude and phase monitor (MAPM) with a signal-to-noise ratio of 51 dB and 11.0 kHz bandwidth, capable of simultaneously driving and sensing the movement of 1D and 2D electrostatically driven micro-actuators without modifying the chip or its packaging. The operational principle is to electromechanically modulate the amplitude of a high-frequency signal with the changing capacitance of the micro-actuator. MAPM operation is characterized and verified by simultaneously measuring the amplitude and phase frequency response of commercial micromirrors. We demonstrate that the MAPM circuitry is insensitive to complex relationships between capacitance and position of the MEMS actuators, and it is capable of giving real-time read-out of the micromirror motion. Our measurements also reveal and quantify observations of phase drift and crosstalk in 2D resonant operation. Measurements of phase changes over time under normal operation also verify the need for phase monitoring. The open-loop, high-sensitivity position sensor enables detailed characterization of dynamic micro-actuator behavior, leading to new insights and new types of operation, including improved control of nonlinear motion.

## 1. Introduction

Electrostatically driven micro-actuators are used in applications where small size, low power, high speed, and large deflections are priorities. Applications span from mass sensors [1] to miniaturized microscopes [2], light detection and ranging (LIDAR) [3], and optical communications [4]. Micro-actuators used for sensing biological, mass, or chemicals would benefit from operating resonantly to achieve larger deflection and higher signal-to-noise ratio (SNR). Microelectromechanical system (MEMS) mirrors used in scanning or shuttering applications are driven resonantly to maximize the speed and reach, while minimizing driving voltages. This is because MEMS resonators are typically underdamped, which gives them a resonant amplitude gain at the resonance frequency. However, resonant drive also leads to large phase gradients as a function of frequency. This means that resonantly driven micro-actuators that experience minute drifts in frequency, e.g., due to environmental changes such as temperature variations, have negligible changes in amplitude, but large changes in phase. High-resolution applications of resonantly driven micro-actuators, therefore, require precise measurements of phase.

Previous solutions for tracking the position of MEMS actuators include integrated sensors, such as two-sided MEMS mirrors [5] or designated sensing structures [6]. Other techniques, such as electromechanical frequency modulation [7] and electromechanical amplitude modulation [8], are hampered by large size, limited bandwidth, and low signal-to-noise ratio in their conventional implementations. Previous two-axis combdrive motion sensors [9] are able to monitor two axes of motion by sensing on one side of each axis. However, this approach sacrifices space on-chip and fails to capture the complete motion of the micro-actuator.

In this paper, we describe a sensor, the MEMS amplitude and phase monitor (MAPM), that provides open-loop, real-time, high-bandwidth, and high-SNR position information. The system can be used with many standard MEMS designs without requiring on-chip circuitry or additional electrical connections. The high sensing frequency and high gain of the MAPM sensor circuit enable large bandwidths and accurate operation to overcome the presence of large parasitic capacitance, because only time-varying capacitances are measured. The measurement circuit yields dynamic information for real-time characterization of nonlinear behavior. The ability to measure the motion behavior on all four sides with high fidelity reveals high-resolution information about mirror movement and allows us to characterize multiple modes of 2D mirrors. This information is important for advanced operations, e.g., interferometric measurements and highly nonlinear motion, and it cannot be obtained via conventional methods such as using a single-position sensing detector (PSD).

The paper is organized as follows: first, we describe the basic design and operation of the MAPM system. Then, we experimentally verify its operation when monitoring 1D motion of micromirrors. The experiments are then extended to 2D operation, which prove the ability of MAPM to monitor complex behavior including crosstalk and combinations of rotation and linear translation.

## 2. MAPM Design and Operation

**Figure 1:**
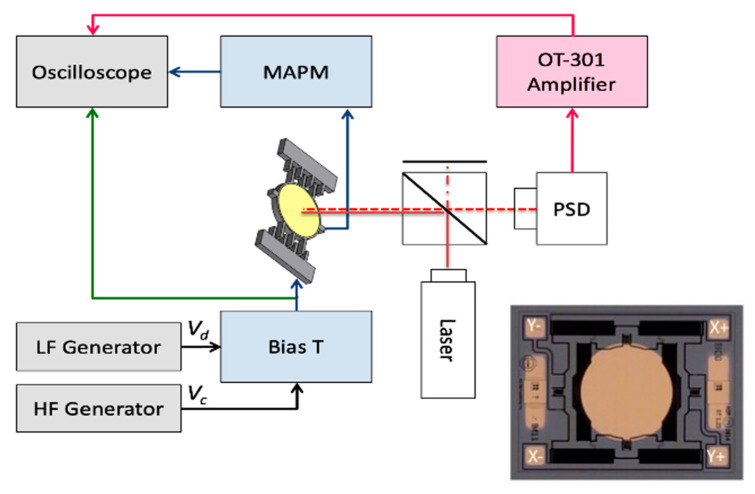
Block diagram of the MAPM experimental setup showing the HF carrier Vc and LF drive Vd combined and sent to one combdrive (Y+) to drive the mirror. To verify operation and calibrate the MAPM, the mirror position is also measured by a PSD. A beam splitter is used to minimize the space between the mirror and PSD. The insert shows the commercial, gimbaled 2D micromirror used in experimental verifications. A voltage applied to the electrode marked Y+ makes the mirror rotate clockwise about the vertical axis, while Y− gives counterclockwise rotation. The same applies for X+ and X− on the horizontal axis.

The MAPM sensor in the 1D setup is depicted in Figure 1 (see Supplemental Material and [10]). The MEMS mirror was biased by a voltage supply (HP6236B) and driven with a low-frequency (LF) generator (Agilent 33120A, Santa Clara, USA). The capacitance of the actuator was monitored at frequencies well above the resonance frequency of the MEMS, using a high-frequency (HF) generator (Keysight 33210A, Santa Rosa, USA). The mirror in the 2D setup was biased by a voltage supply (HP6236B), driven with LF generators (Keysight 33210A, Santa Rosa, USA, Agilent 33120A, Santa Clara, USA), and monitored by two two-channel HF generators (KKMoon DDS, Zhengzhou City, China). There was one MAPM sensor in the 1D setup and four in the 2D setup. The small HF sensing signal and the larger LF drive signal for each actuator were combined on a bias tee that also isolated the signal generators from cross signal feedback. The MEMS actuator was a gimballed 2D commercial micromirror (AG Microsystems, Fremont, USA), shown in the insert of Figure 1. The circular mirror was 1 mm in diameter (d_mirror_) and actuated by four voltage-controlled, electrostatic combdrives with a 5 µm gap (*x*), 30 µm thickness (*y*), and 150 µm overlap (*z*), as described in Table 1. In the 1D experiments, the mirror was driven on one side by one combdrive (Y+), and the MAPM signal was picked up on the opposite comb (Y–). In the 2D experiments, the mirror was actuated and measured simultaneously on all four combdrives. 

The position of an electrostatically driven comb actuator [11,12] can be directly related to the capacitance in the region where combs overlap. During operation, the time-varying voltage drive signal sent to the mirror’s stator combs pulls the rotating combs out of plane, causing the mirror to rotate about its central axis. Using a simple parallel-plate approximation, the capacitance *C_mems_(θ)* can be expressed as follows [13]:

(1) $$C_{mems}\left(\theta \right)=\frac{2N\epsilon }{x}\cdot A\left(\theta \right),$$

where *N* is the number of comb fingers, ϵ is the absolute permittivity of the dielectric material being used, *x* is the gap between two nearest-neighbor comb fingers, and *A(θ)* represents their changing overlap area. In general, the overlap area can also be a function of motion in other degrees of freedom. The approximate expression in Equation (1) ignores fringing fields. This is a good approximation for most MEMS actuators at small angular deflections where the contribution of fringing fields to the total capacitance is negligible. For large angular deflections that make the overlap area small, i.e., the combs of the actuators rotate out of engagement with each other, the fringing fields become the dominant contribution, and Equation (1) is no longer valid. However, the fact that it is valid at small angles means that measurements of capacitance give the correct zero crossings and, therefore, correct phase information. The amplitude measurements are repeatable, and the amplitude can be inferred, but with less precision than the phase; hence, calibration might be necessary. This is an acceptable tradeoff, because phase uncertainty is a bigger problem than amplitude uncertainty in resonantly driven MEMS.

To measure the time-varying capacitance, we measure the ground current as shown in Figure 1. For simultaneous measurements of the current from several (up to four for the 2D setup) MEMS capacitors, all total currents are summed on the input of a transimpedance amplifier, and the corresponding output signals are separated by narrow-band filters matching the HF drives of the separate MEMS capacitors.

The current corresponding to the *N*-th MEMS capacitor can be expressed as follows [14]:

(2) $$I_{CN}\left(t\right)=\frac{V_{CN}\left(t\right)}{Z_{CN}},$$

where *V_CN_(t)* is the voltage, and *Z_CN_* is the impedance of the *N*-th combdrive.

The impedance of each combdrive depends upon the parasitic capacitance CparN, the MEMS capacitor *Cmems_N_* (which changes due to the changing overlap area), and the HF carrier signal’s frequency *ω_N_*.

(3) $$Z_{CN}\left(t\right)=\frac{1}{j\omega_{N}\left(C_{memsN}\left(t\right)+C_{parN}\right)}.$$

The inputs to the MAPM from the mirror in the 1D and 2D setups are first sent through high-pass filters (HPF). Then, each modulated HF signal is isolated by a unique bandpass filter (BPF). The BPFs have similar architectures in the 1D and 2D experimental setups. The 1D BPF is tuned to 1.0 MHz, and, in the 2D case, the BPFs are set to 1.0 MHz, 1.5 MHz, 2.0 MHz, and 2.5 MHz. We avoid the effect of the parasitic capacitances because we filter out the large static signal due to *C_parN_* for each channel, and we directly measure the dynamic capacitance *C_memsN_* current by isolating the changing amplitude of the current from the constant amplitude HF signal of each channel using an envelope detector. To measure each axis in the 2D setup, a differential amplifier takes the absolute difference between the magnitude of each LF sensed signal from each side of each axis, translating the four LF signals (X+, X−, Y+, and Y−) into two difference signals (X*_diff_* and Y*_diff_*), resulting in a total of six MAPM output signals for each drive frequency in the 2D case.

In both experimental setups, the MAPM signals are verified by simultaneously measuring the 1D and 2D mirror motion on a PSD (On-Trak 2L10SP, Irvine, USA) as depicted in Figure 1. Using a PSD is a proven method for measuring MEMS mirror movement [15]. In addition to the PSD and the laser (JDS Uniphase HeNe Laser 1580P, Milpitas, USA), a beam splitter is used to minimize the distance between the mirror and the PSD to allow large scan angles. The PSD uses a differential current to determine spot position and an amplifier (On-Trak OT-301, Irvine, USA) that sends a normalized voltage signal to the oscilloscope (Keysight DSOX3054A, Santa Rosa, USA). The PSD voltage signal *V_out_(t)* is related to the laser beam position *x(t)* by the screen width *w_s_* and normalized voltage range *V_r_* [16].

(4) $$x\left(t\right)=\frac{V_{out}\left(t\right)}{V_{r}}\cdot w_{s}.$$

Both the PSD amplifier and the MAPM output signals are sent to an oscilloscope to measure the amplitude of the voltage signals. The time delay between the first zero crossing of the LF drive signal and the PSD gives the phase delay between the two. The phase delay is similarly measured using the oscilloscope for the MAPM output. 

The frequency response and bandwidth were measured for each of the channels in the 1D and 2D setups, while the MAPM was disconnected from the mirrors and driven with a bandwidth characterization signal. The input signal to the MAPM used to characterize the sensor bandwidth was a varying-frequency (0–30.0 kHz), 0.335 Vpp LF signal with an amplitude modulation of 4% onto each of four equally spaced HF channels between 1.0 MHz and 2.5 MHz signals at 0.758 Vpp, with 0 Vdc bias. The signal at each one of the MAPM output channels was then compared to the bandwidth characterization input signals to measure the amplitude and phase frequency response of the MAPM circuitry. The MAPM sensor bandwidth, independent of the mirrors, for all channels in the 1D and 2D setups was determined to be 11.0 kHz.

The SNR ratios of all seven MAPM outputs (one in the 1D setup and six in the 2D) were measured using an HP400E wide-band voltmeter. The magnitude of the MAPM output signal of each rotor–stator pair and the differential output signal of each axis were measured with the mirror driven resonantly to produce the largest output signal A*_max_* on each axis, and then compared to the MAPM output signals with only the HF signals and no LF signals driving the mirror to produce the minimum output signal A*_min_* for each axis. The SNR was then calculated [17].

SNR(dB) = A*_max_*(dB) − A*_min_*(dB). (5)

In the 1D setup SNR measurement, the mirror was driven with an HF signal (0 Vdc, 0.167 Vpp, 1.0 MHz) for both measurements and an LF signal (49.4 Vdc, 15.3 Vpp, 896.0 Hz) for the resonant measurement. The MAPM SNR was measured to be 42.55 dB on average in the 1D setup. To measure the SNR of each side and axis in the 2D MAPM, each side of the mirror was driven with one of four specific HF signals (1.0–2.5 MHz) at 0.758 Vpp and 0 Vdc for all measurements, plus an LF drive signal for the resonant case. The LF drive signal for the *x*-axis measurements was 20.4 Vpp and −35.2 Vdc at 876.0 Hz. The LF drive signal for the *y*-axis measurements was 20.1 Vpp and −35.2 Vdc at 1077.0 Hz. The measured four-channel MAPM SNR was 48.385 dB for X+ and 47.99 dB for X−, while the Y+ was 28.025 dB and Y− was 23.895 dB with a bandwidth of 11.0 kHz for each of the four channels. The SNR of the *x*-axis differential signal (X*_diff_*) was 50.825 dB, while it was 27.185 dB for the *y*-axis (Y*_diff_*). The difference in SNR measurements between axes was expected, as the geometry of the combs for each axis was different.

## 3. 1D Phase and Amplitude Frequency Responses

**Figure 2:**
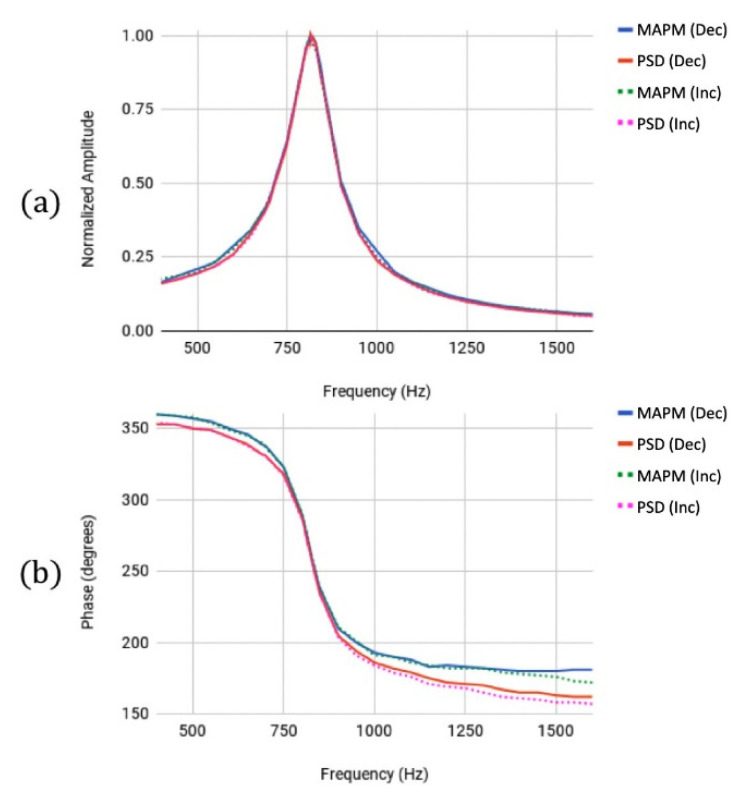
(**a**) Normalized amplitude responses measured by the MAPM (blue/green) and PSD (red/magenta) for low drive voltages. Both the decreasing amplitude frequency response (solid lines) and the increasing amplitude frequency response (dashed lines) have a maximum peak at 817.0 Hz with a Q of 7.8 for both sensors. (**b**) Phase responses measured by the MAPM (blue/green) and PSD (red/magenta). Both the decreasing (solid lines) and the increasing phase frequency response (dashed lines) show a steep phase gradient at 817.0 Hz.

To demonstrate the MAPM’s ability to measure mirror motion for both linear and nonlinear behavior, we used MAPM and PSD to simultaneously record the phase and amplitude frequency responses of the MEMS mirror at a low drive voltage signal (35.3 Vdc and 334 mVpp) and a high drive voltage signal (49.4 Vdc and 15.3 Vpp), decreasing and increasing driving frequencies from 400.0 Hz to 1600.0 Hz. To compare the MAPM and PSD amplitude frequency response measurements for the linear, low drive voltage case, the amplitude measurements were normalized for each sensor by dividing each recorded amplitude by the maximum recorded amplitude from each sensor, which was at 817.0 Hz for both. The normalized amplitude and phase, plotted in Figure 2, demonstrate that the MAPM and PSD were in good agreement for both increasing and decreasing frequency scans.

**Figure 3:**
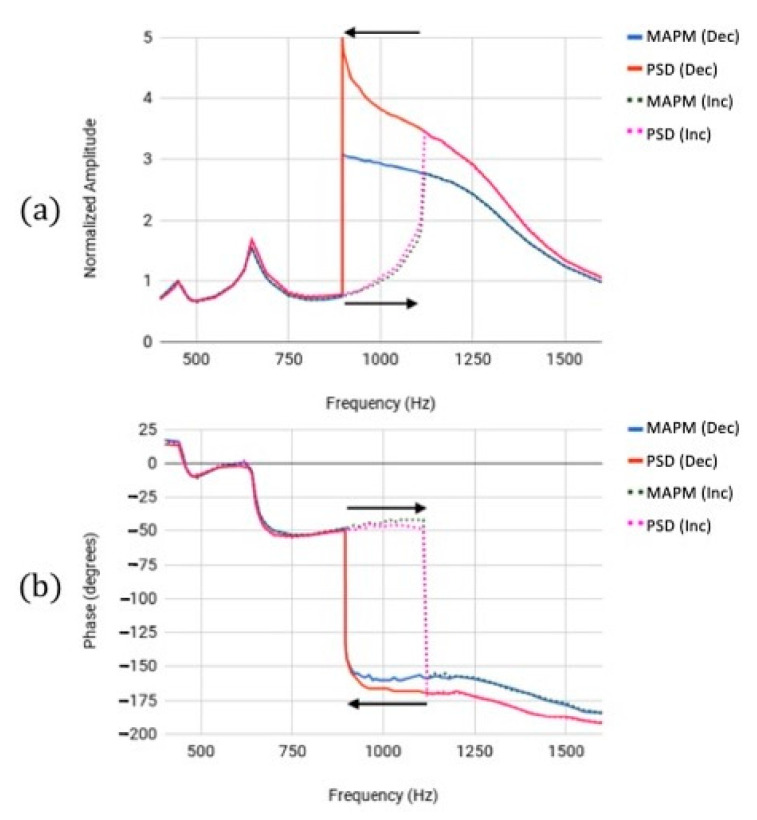
(**a**) Normalized MEMS amplitude frequency responses measured by the MAPM (blue/green) and PSD (red/magenta) for the high drive voltage case. The amplitude responses for the decreasing frequency scan (solid) for both sensors revealed peaks at 450.0 Hz and 650.0 Hz, and a sharp drop at 895.6 Hz. Amplitude responses for the increasing frequency scan (dashed) showed peaks at 450.0 Hz, 650.0 Hz, and 1120.0 Hz. (**b**) Phase responses measured by MAPM (blue/green) and PSD (red/magenta). Phase responses for the decreasing frequency scan (solid) showed sharp changes in phase at 450.0 Hz, 650.0 Hz, and 895.6 Hz. Both phase responses for the increasing frequency scan (dashed) showed steep phase gradients at 450.0 Hz, 650.0 Hz, and 1120.0 Hz.

For high drive voltages (49.4 Vdc and 15.3 Vpp), the amplitude measurements were normalized as follows: at 450.0 Hz, a mechanical swing angle of 0.55° was measured. The maximum comb overlap area change from 4500 µm^2^ to 0 µm^2^ occurred at a mechanical swing angle of 0.568°. Consequently, it is only at frequencies below 450.0 Hz that the overlap area change was the main contributor to the measured capacitance voltage amplitude. Therefore, the amplitude measurements depicted in Figure 3a were normalized to the recorded amplitude at 450.0 Hz for each sensor.

Sharp changes in amplitude were observed at 450.0 Hz and 650.0 Hz, and the peak resonant frequency of 895.6 Hz was measured in the decreasing frequency scan with both sensors as plotted in Figure 3a. The difference in the maximum amplitude measured by the PSD and MAPM was due to the reduced change in the capacitance values as the rotor comb fingers got further from the stator comb fingers, because the change in capacitance was due to fringe capacitance once the comb fingers were no longer overlapping. For the increasing frequency scan plotted in Figure 3a, peak amplitudes were measured with the MAPM and PSD at 450.0 Hz, 650.0 Hz, and 1120.0 Hz. The phase responses measured by the MAPM and PSD also showed sharp gradients at corresponding frequencies for both increasing (450.0 Hz, 650.0 Hz, 1120.0 Hz) and decreasing (450.0 Hz, 650.0 Hz, 895.6 Hz) frequency scans, as seen in the phase frequency responses plotted in Figure 3b. The observed shift in maximum response for increasing and decreasing frequency sweeps was most likely due to mechanical nonlinearity of the spring. The observed behavior is consistent with a softening Duffing spring [18]. The smaller peak amplitudes recorded at the resonant frequencies of 450.0 Hz and 650.0 Hz in Figure 3 occurred due to higher-order harmonic behavior impacting the overall amplitude signals. The higher-order harmonics were measured (Spectran V2 [19]) when the mirror was driven at a high drive voltage (49.4 Vdc and 15.3 Vpp) at 450.0 Hz and 650.0 Hz.

While the amplitude normalization technique employed in Figure 3a allows us to verify that the PSD and MAPM measure relative maxima at specific frequencies, it is important to note that these sensors measure two different phenomena, resulting in the differences observed in the values measured by each sensor for the same axis. The MAPM measures capacitance, and the PSD measures the reflected location of a spot of light; thus, the relative amplitudes differ. The direct relationship between the overlap area and spot location becomes incomplete as the fringe capacitance, which is negligible while the comb fingers are interdigitated, becomes the dominant contribution to the total capacitance when the mirror swings through its maximum angle at 817.0 Hz because the comb fingers are no longer interdigitated. The results illustrate the point that the MAPM accurately measures phase, while accurate amplitude measurements require calibration. 

## 4. MEMS Mirror Phase Stability

**Figure 4:**
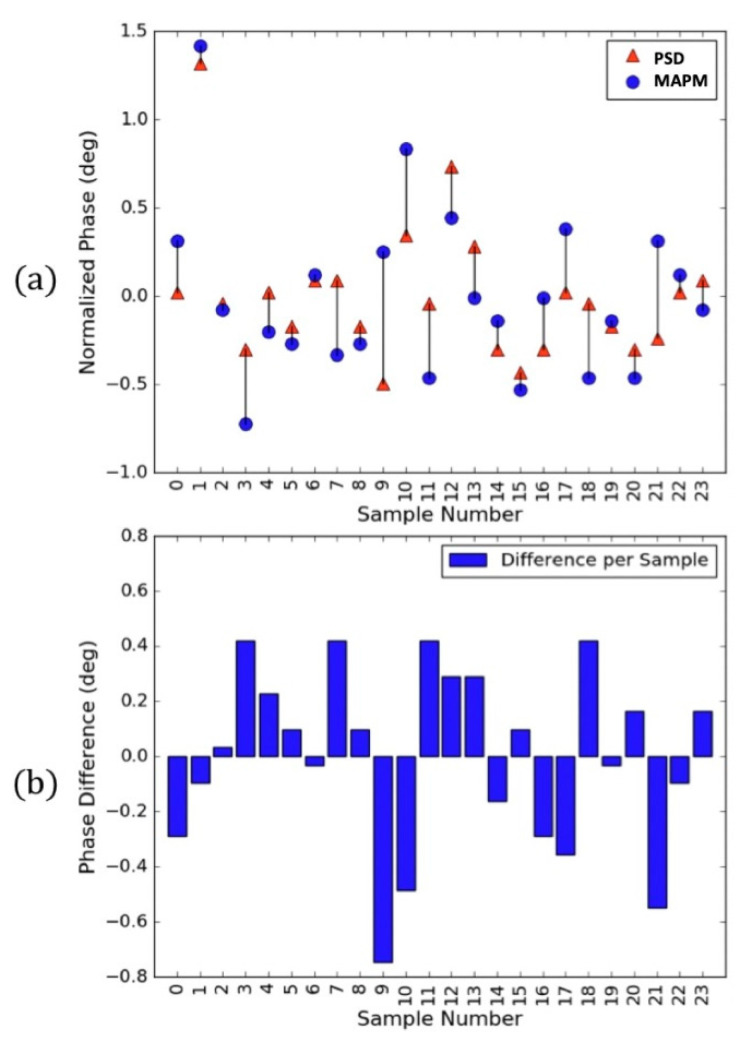
(**a**) The 48 MEMS mirror phase deviations as measured by the MAPM (circles) and PSD (triangles). (**b**) Normalized phase measurement differences between the MAPM and PSD at each point in (**a**) with an average difference of 0° and standard deviation of 0.3197°.

To demonstrate the importance of monitoring the mirror phase, 24 random phase samples over the course of 30 min were observed while driving the mirror with an LF signal (49.4 Vdc, 15.3 Vpp, 897.0 Hz) and an HF signal (0 Vdc, 0.163 Vpp, 1.0 MHz). Each of the 24 phase measurements was captured by comparing the time difference (∆*t*) between the zero-crossing of the input drive signal and the zero-crossing of the output signals of both sensors. All 48 phase measurements (Figure 4) used these time differences to calculate the phase delay *φ* as related by Equation (6) [20], where *f* is the frequency of the measured signal.

(6) $$\varphi =360^{o}\cdot f\cdot \Delta t.$$

Due to the different inherent phase delays introduced by the circuitry of the PSD and MAPM, the phase values depicted in Figure 4a were normalized by taking the average measured phase of each system’s 24 measurements and subtracting that average from each measured phase. The phases depicted in Figure 4b are the differences between the normalized PSD and MAPM phase measurements, which are the same as the black lines connecting the MAPM and PSD measurements in Figure 4a, but moved to the *x*-axis to directly compare the measured phase deviations.

## 5. 2D MAPM Experiments

Measurements made using 4MAPM demonstrated a maximum SNR of approximately 50 dB, a bandwidth of 11.0 kHz, and the ability to characterize the phase and amplitude frequency response of all four sides of a 2D MEMS mirror through the same leads used to operate it. The frequency response of each side of the *x*- and *y*-axis of the other 2D MEMS mirror was measured in the 2D experimental setup with a four-channel MAPM (4MAPM) configuration by driving the *x*- and *y*-axes in a push–pull configuration. One side (X+) was driven with a 20.4 Vpp sinusoidal drive signal at −35.2 Vdc bias voltage, while the other (X−) was driven with the same signal, but 180° out of phase, for a frequency range of 400.0 Hz to 1.5 kHz. Each side of the *x*-axis (X+, X−) was simultaneously monitored with an HF carrier signal at 1.0 MHz and 1.5 MHz, respectively. The frequency response of each side of the *y*-axis (Y+, Y−) was measured with the same LF driving voltage scheme as the *x*-axis, but with HF carrier signals at 2.0 MHz and 2.5 MHz, respectively. 

The 2D PSD and 4MAPM sensor configurations both measured the maximum amplitude response for the *x*-axis at 817.0 Hz, with a secondary resonant amplitude at 1280.0 Hz, verifying the 4MAPM measurements on each side of the *x*-axis. The PSD and 4MAPM both measured the maximum amplitude response for the *y*-axis at 1050.0 Hz, with a secondary amplitude response at 560.0 Hz, verifying the 4MAPM measurements on each side of the *y*-axis.

The differential outputs of the 4MAPM X_*diff*_ and Y_*diff*_ are the absolute differences between the magnitude of each LF sensed signal from each side of each axis. This allows for the amplitude and phase frequency response of each rotor–stator comb pair of each axis to be simultaneously measured and compared to the X and Y output of the PSD using the same driving and carrier signals previously described for measuring the frequency response of each rotor–stator comb pair. The PSD and 4MAPM also measured the maximum phase change for the *x*-axis at 817.0 Hz, with a secondary drop at 1280.0 Hz. The maximum phase change for the *y*-axis was also measured at 1050.0 Hz for both the PSD and the 4MAPM, with a secondary phase jump at 560.0 Hz, verifying that the 4MAPM identified the same resonant frequencies in the phase response measurements for each axis.

**Figure 5:**
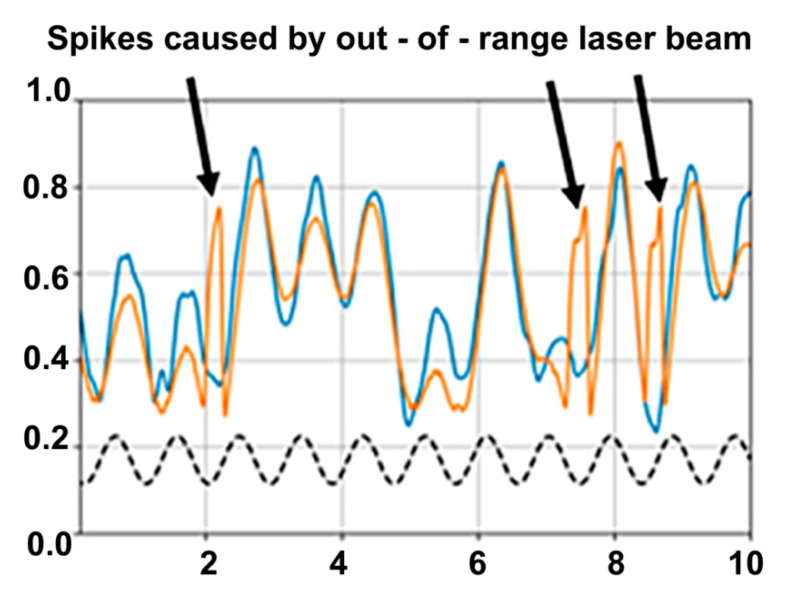
Details of MEMS mirror’s motion while driven at 1100.0 Hz (dash—20 V/div) are recorded by the 4MAPM *y*-axis differential output signal (blue—200 mV/div) and by the *y*-axis signal from the PSD (orange—2.3 V/div). To simplify comparison, the 4MAPM signal is phase-shifted to best match the PSD. The two recorded signals match well except where the laser beam position overshoots the PSD range, leading to anomalous spikes in the PSD signal (arrows).

The on-chip integration of the 4MAPM allows more robust measurements of large and complex deflections than can be achieved practically with the PSD. When the micromirror is driven very hard on resonance, the 4MAPM is able to follow the resulting large and erratic motion, while the PSD gives rise to anomalous spikes in the position signal when the range of motion becomes too large, as shown in Figure 5. At the times the spikes occur, MAPM gives more correct and tractable position values of the mirrors, showing the strength of on-chip measurements versus using a PSD that requires very accurate positioning.

**Figure 6:**
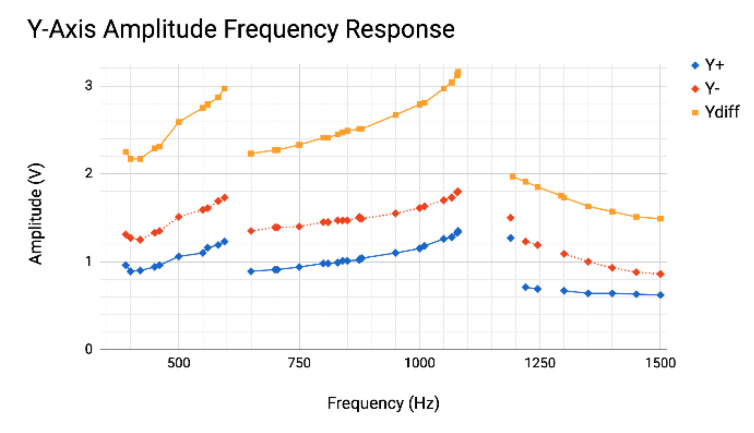
Increasing frequency amplitude response of each rotor–stator comb pair (diamonds) of the *y*-axis and the simultaneously recorded *y*-axis differential signal Y*_diff_* (squares), as measured by the 4MAPM, to demonstrate that the Y− (red) rotor–stator comb pair measured a larger minimum, maximum, and overall average capacitance change versus the Y+ (blue) comb pair.

Nominal voltage outputs from each comb pair and each axis can be compared in the 2D application of the MAPM sensor because all channels were tuned to provide equivalent output voltages. The benefit of calibrated output is clearly demonstrated in Figure 6, which shows that the Y− comb pairs produced larger capacitance voltage amplitudes than the Y+. Likewise, the 4MAPM measured the X− comb pairs produced a larger capacitance voltage amplitude than the X+ comb pairs. These measurements show that the 4MAPM is capable of identifying a comb pair imbalance—possibly due to a fabrication incongruity in the chip, such as one pair of comb fingers overlapping more on one side—which would otherwise be difficult to visualize during chip operation or after installation.

To characterize crosstalk from the *y*-axis movement onto the *x*-axis, the amplitude and frequency of the small crosstalk modulation envelope on the X*_diff_* 4MAPM output signal were measured and compared to the overall amplitude and frequency of the X output of the 4MAPM. The amplitude effect of crosstalk was measured in terms of percent amplitude modulation of the *y*-axis onto the *x*-axis and vice versa while the *x*-axis was driven at 876.0 Hz with 20.4 Vpp and −35.19 Vdc bias, and the *y*-axis was driven at 1077.0 Hz with 20.1 Vpp, and −35.19 Vdc bias. The measured crosstalk amplitude modulation of the *y*-axis onto the *x*-axis was 15.44% and 14.7% according to the 4MAPM and PSD, respectively. The crosstalk amplitude modulation of the *x*-axis onto the *y*-axis was 8.47% and 8.96% according to the 4MAPM and PSD, respectively.

Each axis affects the other differently due to their different geometries. In this particular mirror, the chip was constructed with the *y*-axis embedded inside of the *x*-axis. This means that the *y*-axis is smaller and at the center of the *x*-axis, resulting in a larger effect of the y-axis onto the x-axis motion. By finding matching amplitude crosstalk effects on the motion signal for each respective axis with the PSD and 4MAPM, we can verify not only the ability of the 4MAPM to accurately measure the presence of this effect, but that it is a large—roughly ≥ 10%—amplitude distortion on both axes.

**Figure 7:**
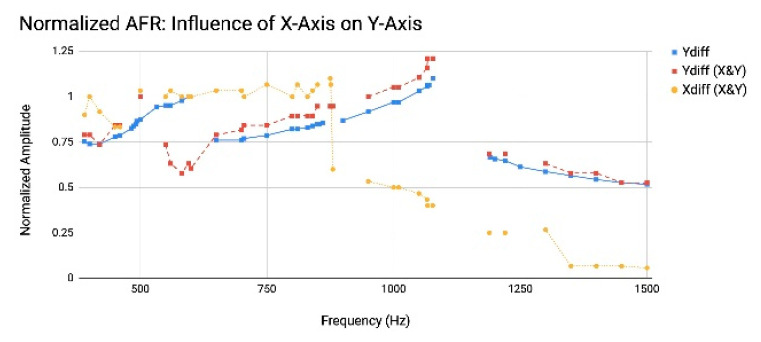
Crosstalk effect on amplitude frequency response (AFR) behavior of *y*-axis, most notably at secondary amplitude response peak evident at 650.0 Hz for the Y*_diff_* output signal when the *y*-axis is driven without driving the *x*-axis (blue), versus the amplitude minimum at 650.0Hz (red) when the *x*-axis (orange) is driven simultaneously.

To identify the impact of driving the mirror on both axes on the frequency response of the mirror, the frequency response of the *y*-axis was measured without driving the *x*-axis and compared to the frequency response of the *y*-axis while simultaneously characterizing the frequency response of the *x*-axis. The frequency response of each axis was measured by increasing the frequency from 400.0 Hz to 1.5 kHz, at 20 Vpp and −35.19 Vdc bias. All four HF signals were supplied to the mirror at 0.8 Vpp and 0 Vdc for both the single- and two-axis frequency response measurements. As shown in Figure 7, when the *y*-axis was driven without driving the *x*-axis, there was a primary peak amplitude response at 1077.0 Hz and a secondary peak at 650.0 Hz. However, when the *x*-axis was driven simultaneously, the secondary peak amplitude response on the *y*-axis turned into a local minimum in amplitude. This 2D frequency crosstalk demonstrates how 2D resonant operation can shift the amplitude frequency and amplitude behavior of one axis due to the operation of the other. The change in frequency behavior between 1D and 2D, observed in Figure 7, demonstrates the necessity of monitoring the motion. 

## 6. Conclusions

By measuring the amplitude and phase of a 2D micro-actuator from all four sides, we quantified the unbalanced behavior and observed the fine details of the scan pattern with the ability to inspect the motion of each rotor–stator comb pair on each axis. We also verified the need for phase monitoring by measuring phase changes over time under normal operation. By using a commercial mirror for our experiments, we highlighted one of the main features of the MAPM sensing technique, i.e., the micromirror chip needs no modifications, and the dimensions of the mirror do not have to be known, making the MAPM sensing technique a very useful and unique tool. MAPM is also able to characterize the amplitude and phase frequency response of each axis with high fidelity without external access or prior knowledge of the micro-actuator dimensions. Additionally, the 4MAPM sensor was also able to identify and simultaneously monitor the nonlinear, unstable phase and crosstalk behavior of a multidimensional micro-actuator driven simultaneously in 2D. The motion and chip structure information accessible with the MAPM has the potential to confer electrostatically driven micro-actuators with the ability to overcome their inherently unpredictable and complex motion behavior. By identifying these multidimensional operation effects, the importance of having the ability to simultaneously measure the behavior of each motional axis for multidimensional micro-actuators becomes apparent in terms of micro-actuator characterization and feedforward error correction. With feedforward error correction, the monitoring capability offered by the MAPM sensor design has the potential to allow the micro-actuator industry to overcome hurdles created by the little-understood, complex behavior identified in this work. The observed complex phenomena occurring during multidimensional operation also open the door for new multidimensional micro-actuator applications, such as capitalizing on crosstalk amplitude modulation to transform traditional scan patterns or communication signals into unique and novel schema. By identifying and quantifying both rotational and linear motions on each axis of motion, detailed characterization of all degrees of motion is achievable, leading to the development of new imaging and display modalities that use MAPM data to correct the images through feedforward software modifications. In addition, error correction can be applied for improved high-resolution operation.

## Figures and Tables

**Table 1:** Mirror and combdrive parameters.

D_mirror_	N_y_	N_x_	Gap (*x*)	Thickness (*y*)	Overlap (*z*)
1 mm	54	75	5 µm	30 µm	150 µm

## Data Availability

Data are contained within the article.

## Supplementary Materials

The following are available online at https://www.mdpi.com/article/10.3390/mi13081360/s1: Figure S1. Block diagram of the MAPM System.

## References

[1] Ramanan A., Teoh Y.X., Ma W., Ye W. (2016). Characterization of a laterally oscillating microresonator operating in the nonlinear region. Micromachines.

[2] Jeong J.W., Kim S., Solgaard O. (2012). Split-frame gimbaled two- dimensional MEMS scanner for miniature dual-axis confocal microendo- scopes fabricated by front-side processing. J. Microelectromech. Syst..

[3] Hofmann U., Senger F., Soerensen F., Stenchly V., Jensen B., Janes J. Biaxial resonant 7mm-MEMS mirror for automotive LIDAR application. Proceedings of the 2012 International Conference on Optical MEMS and Nanophotonics.

[4] Solgaard O., Godil A.A., Howe R.T., Lee L.P., Peter Y.A., Zappe H. (2014). Optical MEMS: From micromirrors to complex systems. J. Microelectromech. Syst..

[5] Sandner A.T.T., Kenda A. Design of an optical position detection unit for fast 2D-MOEMS scanners. Proceedings of the SPIE Optical Systems Design.

[6] Trusov A.A., Shkel A.M. Parallel Plate Capacitive Detection of Large Amplitude Motion in MEMS. Proceedings of the 14th International Conference on Solid-State Sensors, Actuators and Microsystems.

[7] Moore S.I., Moheimani S.O.R. (2014). Simultaneous actuation and sensing for electrostatic drives in MEMS using frequency modulated capacitive sensing. IFAC Proceedings Volumes (IFAC-PapersOnline).

[8] Dong J., Ferreira P.M. (2008). Simultaneous actuation and displacement sensing for electrostatic drives. J. Micromech. Microeng..

[9] Wantoch T.V., Mallas C., Hofmann U., Janes J., Wagner B., Benecke W. Analysis of capacitive sensing for 2D-MEMS scanner laser projection. Proceedings of the SPIE MOEMS-MEMS.

[10] Manosalvas-Kjono S., Quan R., Solgaard O., Sun Z. (2021). Method and Apparatus for Evaluating Electrostatic or Nonlinear Devices. U.S. Patent.

[11] Tang W.C.-K. (1990). Electrostatic Comb Drive for Resonant Sensor and Actuator Applications. Ph.D. Thesis.

[12] Holmström S.T.S., Baran U., Urey H. (2014). MEMS laser scanners: A review. J. Microelectromech. Syst..

[13] Liu C. (2012). Foundations of MEMS.

[14] Zumbahlen H. (2008). Linear Circuit Design Handbook.

[15] Chen H., Chen A., Sun W.J., Sun Z.D., Yeow J.T. (2016). Closed-loop control of a 2-D mems micromirror with sidewall electrodes for a laser scanning microscope system. Int. J. Optomechatron..

[16] On-Trak Photonics Website. https://www.on-trak.com/specchart.html.

[17] Blake R. (2001). Wireless Communication Technology.

[18] Kreyszig E. (1999). Advanced Engineering Mathematics.

[19] Spectran V2 Website. https://www.sdradio.eu/weaksignals/spectran.html.

[20] Ballou G. (2005). Handbook for Sound Engineers.

