# Supplementary material for: Integrated Amplitude and Phase Monitor for Micro-Actuators
Source: Micromachines (Basel). 2022 Aug 20;13(8):1360. doi: 10.3390/mi13081360 (PMC9415144; doi:10.3390/mi13081360)
Supplement: Supplementary file 1 [file micromachines-13-01360-s001.zip › micromachines-1868103-supplementary.pdf]

micromachines

Article

Electrostatic Amplitude and Phase Monitor for Multi-Dimensional Micro-actuators

Sandra N. Manosalvas-Kjono*, Ron Quan and Olav Solgaard

Department of Electrical Engineering, Stanford University, Stanford, California, USA
*Correspondence: kjono@stanfordalumni.org

Abstract: Micro-actuators driven on resonance maximize reach and speed, but due to their sensitivity to environmental factors (e.g., temperature, air pressure, etc.), the amplitude and phase response must be monitored to achieve accurate actuator position. We introduce a MEMS (Microelectromechanical Systems) Amplitude and Phase Monitor (MAPM) with a signal-to-noise ratio of 51dB and 11kHz bandwidth, capable of simultaneously driving and sensing the movement of 1D and 2D electrostatically-driven micro-actuators without modifying the chip or its packaging. The operational principle is to electromechanically modulate the amplitude of a high frequency signal with the changing capacitance of the micro-actuator. MAPM operation is characterized and verified by simultaneously measuring the amplitude and phase frequency response of commercial micromirrors. We demonstrate that the MAPM circuitry is insensitive to complex relationships between capacitance and position of the MEMS actuators and capable of giving real-time read-out of the micromirror motion. Our measurements also reveal and quantify observations of phase drift and cross-talk in 2D resonant operation, and measurements of phase changes over time under normal operation verify the need for phase monitoring. The open-loop, high-sensitivity position sensor enables detailed characterization of dynamic micro-actuator behavior, leading to new insights and new types of operation, including improved control of non-linear motion.

Keywords: combdrive; phase; open-loop; resonance; sensor; capacitance; MEMS; multiple frequency bandpass filters; multiple frequency demodulators

MAPM Utilizing Multiple RF Signals and AM Demodulators

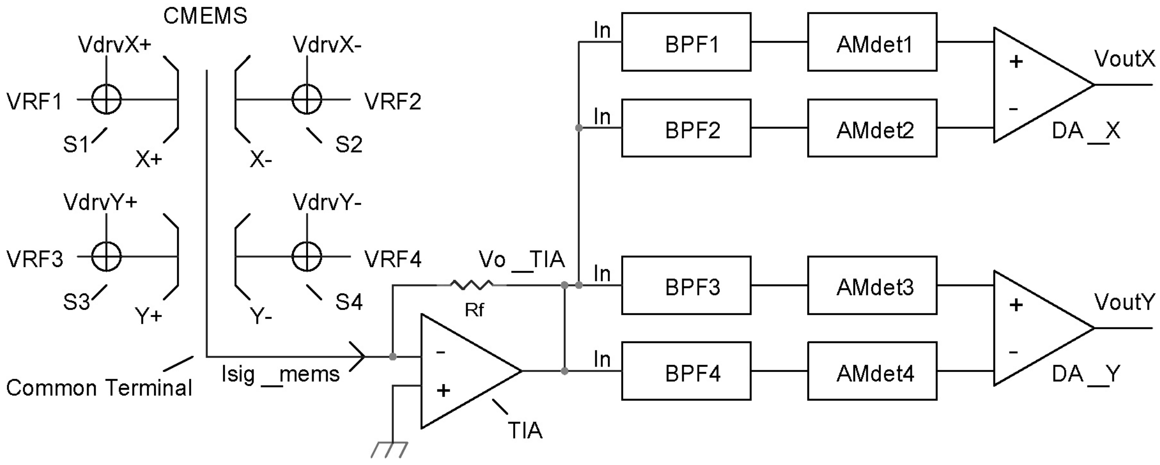

Figure S1. Block diagram of the MAPM System.

The system provides signals VoutX and VoutY for measuring the amplitude and phase of a MEMS scanner. The Common Terminal is connected to the moving X and Y portions of the electrostatic device, with associated capacitive impedances Z_{CX+}, Z_{CX-}, Z_{CY+}, and Z_{CY-} related to the four stator electrodes, X+, X-, Y+, and Y-. Four RF (radio frequency) signals, VRF1, VRF2, VRF3, and VRF4 at 1.0 MHz, 1.5 MHz, 2.0 MHz, and 2.5 MHz are coupled to CMEMS for capacitance measurements related to the moving X and Y portions and the four stator electrodes. The MEMS scanner is driven in the X axis with push-pull signals VdrvX+ and VdrvX- at frequencies < 2 kHz. For the Y axis, CMEMS is driven by push pull signals VdrvY+ and VdrvY- at frequencies < 2 kHz. Also the X and Y push pull signals include a DC bias voltage. The drive signals and RF signals are combined via summing circuits S1 to S4 to the four stator electrodes.

Isig_mems = $I_{CX+}(t) + I_{CX-}(t) + I_{CY+}(t) + I_{CY-}(t) = \frac{V_{RF1}(t)}{Z_{CX+}} + \frac{V_{RF2}(t)}{Z_{CX-}} + \frac{V_{RF3}(t)}{Z_{CY+}} + \frac{V_{RF4}(t)}{Z_{CY-}}$

Signal Isig_mems is fed to transimpedence amplifier, TIA, which provides an output signal voltage Vo_TIA = - (Isig_mems x Rf) where Rf is the feedback resistor of the TIA.

Vo_TIA = $-\left(\frac{V_{RF1}(t)}{Z_{CX+}} + \frac{V_{RF2}(t)}{Z_{CX-}} + \frac{V_{RF3}(t)}{Z_{CY+}} + \frac{V_{RF4}(t)}{Z_{CY-}} \right)$ x Rf

Because the push pull drive signals produce time varying capacitive impedances, the output signal, Vo_TIA, provides four amplitude modulated signals at 1.0 MHz, 1.5 MHz, 2.0 MHz, and 2.5 MHz. To demodulate these amplitude modulated signals separately, Vo_TIA is coupled to four band pass filters, BPF1 centered at 1.0 MHz, BPF2 centered at 1.5 MHz, BPF3 centered at 2.0 MHz, and BPF4 centered at 2.5 MHz. Each band pass filter is fed to a respective amplitude modulation detector (e.g., envelope detector). AM detectors, AMdet1, AMdet2, AMdet3, and AMdet4 provide signals related to the capacitances associated with the X+, X-, Y+, and Y- stator electrodes. To provide signal indicative of the X and Y motions, differential amplifiers DA_X and DA_Y are connected to the AM detectors as shown. VoutX and VoutY from the differential amplifiers provide signals indicative of the X and Y motions.
